# Longitudinal Evaluation of AFP and CEA External Proficiency Testing Reveals Need for Method Harmonization

**DOI:** 10.3390/diagnostics13122019

**Published:** 2023-06-09

**Authors:** Nathalie Wojtalewicz, Laura Vierbaum, Anne Kaufmann, Ingo Schellenberg, Stefan Holdenrieder

**Affiliations:** 1 INSTAND e.V., Society for Promoting Quality Assurance in Medical Laboratories, Ubierstr. 20, 40223 Duesseldorf, Germanys.holdenrieder@tum.de (S.H.); 2 Institute of Bioanalytical Sciences (IBAS), Center of Life Sciences, Anhalt University of Applied Sciences, Strenzfelder Allee 28, 06406 Bernburg, Germany; 3 Institute of Laboratory Medicine, Munich Biomarker Research Center, Deutsches Herzzentrum München, Technische Universität München, 80636 Munich, Germany

**Keywords:** external quality assessment, proficiency testing, tumor marker, CEA, AFP, alpha-fetoprotein, carcinoembryonic antigen, INSTAND, assay harmonization

## Abstract

The glycoproteins alpha-fetoprotein (AFP) and carcinoembryonic antigen (CEA) have long been approved as biomarkers for diagnosing and monitoring tumors. International Reference Preparations (IRPs) have been around since 1975. Nevertheless, manufacturer-dependent differences have been reported, indicating a lack of harmonization. This paper analyzes data from 15 external quality assessment (EQA) surveys conducted worldwide between 2018 and 2022. The aim was to gain insight into the longitudinal development of manufacturer-dependent differences for CEA and AFP. In each survey, participating laboratories received two samples with different tumor marker levels. Inter- and intra-assay variability was analyzed and the mean 80% and 90% of the manufacturer collectives were compared to the evaluation criteria of the German Medical Association (RiliBÄK). The median EQA results for CEA revealed manufacturer-dependent differences between the highest and lowest collective of up to 100%; for AFP, the median differences mostly remained below 40%. The coefficients of variation were predominantly low for both markers. We concluded that the current assays for AFP and CEA detection are better harmonized than previously reported. The assays displayed a good robustness; however, a narrowing of the current assessment limits in EQA schemes could further enhance the quality of laboratory testing.

## 1. Introduction

Tumor markers have been established as useful indicators which aid in tumor diagnosis or prognosis (e.g., alpha-fetoprotein (AFP) in hepatocellular carcinoma (HCC) [1,2,3,4]), facilitate treatment decisions (e.g., human epidermal growth receptor 2 in breast cancer (reviewed in [5])), and monitor disease progression and the effectiveness of treatment (e.g., carcinoembryonic antigen (CEA) in colorectal cancer (CRC) (reviewed in [6,7,8])). The glycoproteins AFP and CEA have long been approved as biomarkers, with International Reference Preparations (IRPs) having been available since 1975 [9]. Even though these reference standards have been around for decades, multiple studies have reported method- or manufacturer-dependent differences for both markers [10,11,12,13,14,15]. The Society for Promoting Quality Assurance in Medical Laboratories (INSTAND) is a German, non-profit interdisciplinary scientific medical society. In 2005, we observed a manufacturer-dependent bias of up to 23% for AFP and up to 85% for CEA [13]. Furthermore, several research groups have reported method bias for both CEA [10,15] and AFP [16]. In the case of CEA, Zhang et al. found a missing matrix-dependent harmonization of four different test systems, even for the first IRP 73/601 [15]. These results substantiate the fact that the same assay should always be used to measure all of a patient’s samples [12,17].

In this paper, we re-evaluate the development of the quality of laboratory testing between 2018 and 2022 for the tumor markers CEA and AFP. We have chosen to focus on both markers, since EQA data analysis focuses on the analytical aspects and there are long-established IRPs available. We also review the scatter of values observed for individual manufacturer collectives in relation to the assessment limits for the EQA schemes of these two tumor markers as stated by the German Medical Association [18].

## 2. Materials and Methods

### 2.1. Sample Materials—Properties and Preparation

Human serum pools were used as EQA sample matrix. Samples were stabilized by adding 0.02% sodium azide. No other synthetic substances were added. The final tumor marker concentrations for the individual EQA surveys were achieved by spiking with non-synthetic tumor markers from tumor tissue cell lines. A commutability study is pending, but a study that used similar sample material deemed a lack of commutability due to the minimal manipulation of the sample material unlikely [19]. Homogeneity and stability of each sample batch were declared and issued by the manufacturer. The liquid samples were stored at 2 °C to 8 °C until shipment in the EQA survey.

### 2.2. EQA Procedure

The INSTAND EQA scheme for tumor marker detection is offered globally six times a year (T1 to T6). Two different concentrated samples per survey are sent to the participating laboratories. The participants are asked to report their quantitative results for CEA and AFP, as well as other tumor markers, and to provide INSTAND with information on the respective device, reagent, and method used.

As no reference method procedure is available, the consensus value of manufacturer-specific collectives, calculated with algorithm A ([20] Section C3), serves as the target value for the evaluation of the participant results and for the laboratory certification. The EQA passing criterion for CEA and AFP was ±24% of the consensus value over the entire evaluation period in accordance with the guidelines of the German Medical Association (RiliBÄK) [18].

### 2.3. Data Analysis and Statistics

In this study, the CEA and AFP results were analyzed for the EQA surveys 2018-T1 to 2022-T1. Due to the large number of EQA surveys, only the data from the three annual EQAs with the largest number of participants (T1 (January), T3 (May), and T6 (October)) were evaluated (Table S1). Thus, a total of thirteen CEA and AFP surveys were analyzed. Results from individual participants that involved sample mix-ups or reporting errors were excluded from the analysis. This applied to 15 results for CEA and AFP, respectively.

The EQA data were analyzed in a manufacturer-dependent manner (Table S2). Six manufacturer collectives (number of participants ≥ 11) were included in the analysis of the CEA results, while there were five collectives (number of participants ≥ 8) for AFP. The distributions of results are shown as box plot diagrams over time. The SI collective comprises four manufacturer sub-collectives under the consolidation of Siemens. In some EQA surveys, we observed a multimodality in the SI collective (Table S2, Figures S1 and S2).

For the different surveys, the median of the collectives was normalized to the overall median of the respective survey to gain an impression of the standardization of the manufacturer-based results. Due to the multimodality of the SI collective, the normalized median was shown for the two larger SI sub-collectives BG and DG, while we showed all SI results in the general boxplot analysis to get a better understanding of the value distribution of this manufacturer. 

The coefficients of variation (CVs) were calculated to quantify the scatter within the manufacturer collectives. For SI, the two sub-collectives BG and DG were again represented. Manufacturer-dependent values that scattered further than the 1.5-fold interquartile range, the width between the 25th and 75th percentiles, were defined as outliers and excluded before calculating the CVs. 

The manufacturer-dependent value distribution is shown in relation to the EQA success criteria in order to obtain an overview of the inter-laboratory performance quality of CEA and AFP detection. According to the guidelines of the German Medical Association (RiliBÄK), the EQA assessment limit is ±24% of the collective target value for CEA and AFP, respectively [18]. The 10th and the 90th percentiles as well as the 5th and the 95th percentiles of the value distribution are shown for both tumor markers and for each manufacturer collective and sample. In this study, the median serves as reference as it represents the robust mean very well.

Basic statistical analyses were performed using jmp 16.0.0 from SAS Institute (Cary, NC, USA).

### 2.4. Generation of Images

The overlay images were generated using version 2.10.8 of the Gnu image manipulation software.

## 3. Results

The EQA data for CEA and AFP detection were analyzed for thirteen surveys—T1, T3, and T6 between 2018 and 2022. The participating laboratories reported a total of 8287 results for CEA and 5306 for AFP.

**Figure 1:**
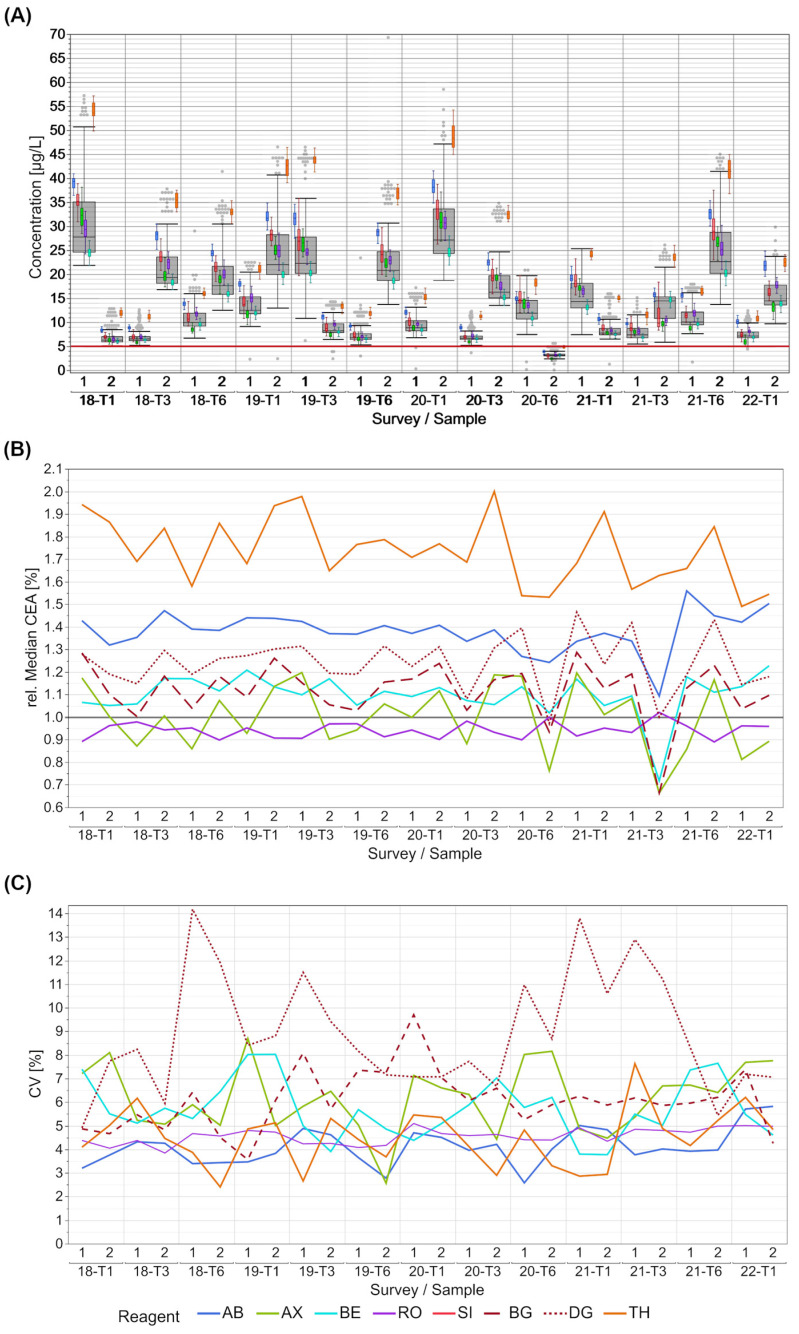
Manufacturer-dependent analysis of EQA results for CEA levels for all results (**A**), a comparison of manufacturer-dependent median differences in comparison to the overall median (**B**), and evaluation of manufacturer-dependent CVs (**C**), shown from 2018 to the beginning of 2022. Data of two samples per survey are shown. The grey boxes display all results for the respective sample, and the distributions of specific manufacturer-based collectives are illustrated as smaller, colored box plots in overlay with the total results (blue: AB, green: AX, cyan: BE, violet: RO, bright red: SI (only present in figure (**A**), dark red: BG, dark red dotted: DG, orange: TH). The cut-off value of 5 µg/L is marked with a red line in the figure. Grey dots mark the outliers of all results. Outliers were excluded from colored boxes. For all boxes, the whiskers stretch from the 1st quartile − 1.5 * (interquartile range) to the 3rd quartile + 1.5 * (interquartile range). Due to the multimodality of the SI collective, the relative median and the CVs were calculated for the two larger SI sub-collectives BG and DG.

The distribution of the CEA results shows manufacturer-dependent concentration differences; however, the scatter within the manufacturer collectives is quite low (Figure 1A). Recurring patterns can be observed in the differences between collectives for the different surveys over the years. For most samples, the lowest values were obtained by participants of the RO collective, with some exceptions for samples with concentrations close to the cut-off value of 5 µg/L. This value most frequently corresponds to the 95th percentile of healthy individuals and is therefore used in clinical decision making. Here the AX collective showed the lowest results. The collective with the highest values for all samples was TH. The values of this collective showed no overlap with those of other collectives until 2020-T6. From this point onwards, it was in slightly better alignment with some manufacturers as long as the median total CEA concentration was below 20 µg/L (Figure 1A).

These observations are also reflected in the relative collective medians of CEA, normalized to the total median of the sample results. The normalized median differences amounted to 100% when the collectives with the lowest and highest values are compared (Figure 1B). The normalized median of the collectives AB, AX, BE and the SI sub-collectives BG and DG dropped conspicuously for individual samples, e.g., sample 2 for EQAs 2020-T6 and 2021-T3, in contrast to the median of the RO collective.

The CVs of the manufacturer collectives are predominantly low for all EQA samples (Figure 1C). With the exception of the DG sub-collective, the CVs of all collectives remained below 10% for both samples over the thirteen EQAs. The AB and RO collective did not exceed a maximum CV of 6%. The DG sub-collective showed increased CVs of over 10% and up to 14% several times. In this EQA survey, the SI sub-collective DG showed noticeable higher values for CEA than the others (Figure S1).

**Figure 2:**
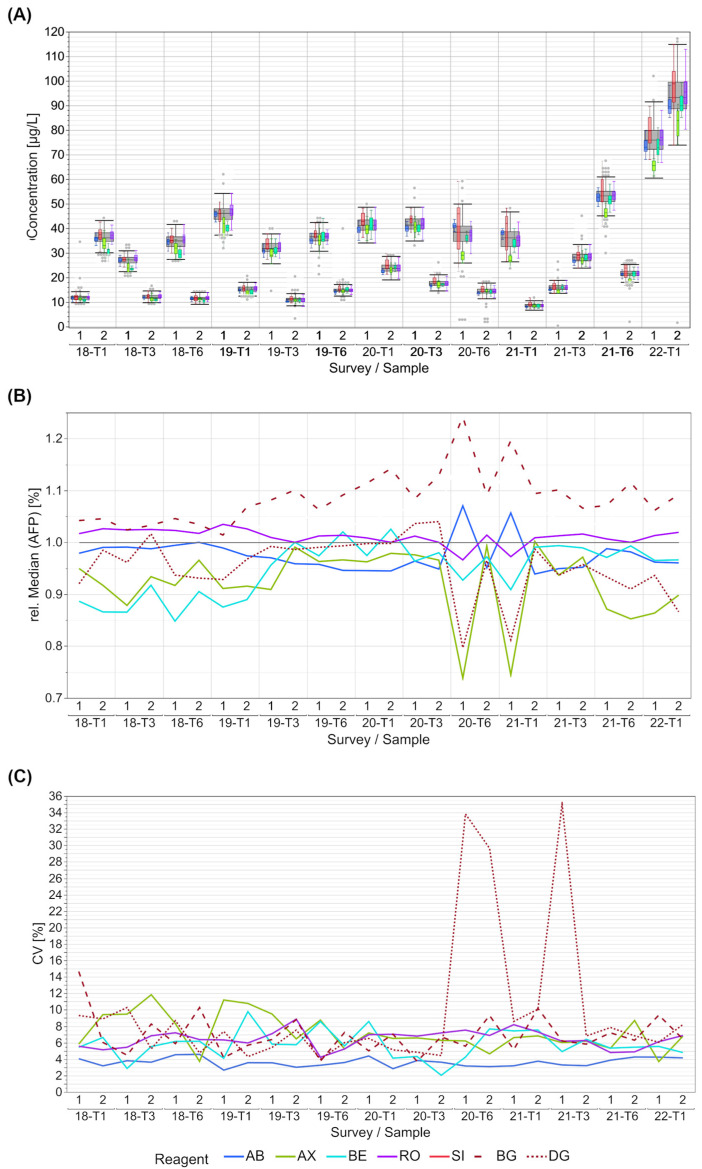
Manufacturer-dependent analysis of EQA results for AFP levels for all results (**A**), a comparison of manufacturer-dependent median differences in comparison to the overall median (**B**), and evaluation of manufacturer-dependent CVs (**C**), shown from 2018 to the beginning of 2022. Data of two samples per survey are shown. The grey boxes display all results for the respective sample, and the distributions of specific manufacturer-based collectives are illustrated as smaller, colored box plots in overlay with the total results (blue: AB, green: AX, cyan: BE, violet: RO, bright red: SI (only present in (**A**)), dark red: BG, dark red dotted: DG). Grey dots mark the outliers of all results. Outliers were excluded from colored boxes. For all boxes, the whiskers stretch from the 1st quartile − 1.5 * (interquartile range) to the 3rd quartile + 1.5 * (interquartile range). Due to the multimodality of the SI collective, the relative median and the CVs were calculated for the two larger SI sub-collectives BG and DG.

The distribution of the AFP results was well-harmonized between the collectives for most samples (Figure 2A). Schemes 2019-T3 to 2020-T3 exhibited a particularly good harmonization of the relative collective medians (Figure 2B). A tendency toward higher values is shown for the BG collective starting with 2019-T3 (Figure S2). For the two SI sub-collectives BG and DG, contrary medians were observed, which is consistent with a larger scatter of the SI collective (Figure 2A). The BE collective had the lowest collective medians between 2018-T1 and 2019-T1; however, it was close to the total median thereafter. Since 2020-T6, the AX and the DG collective often reported lower values compared to the overall results. 

In terms of AFP detection, all of the manufacturer collectives had CVs below 12%, except for samples 1 and 2 in 2020-T6 and sample 1 in 2021-T1 in the DG sub-collective (Figure S2). The AB collective had the overall lowest CV of <5%.

**Figure 3:**
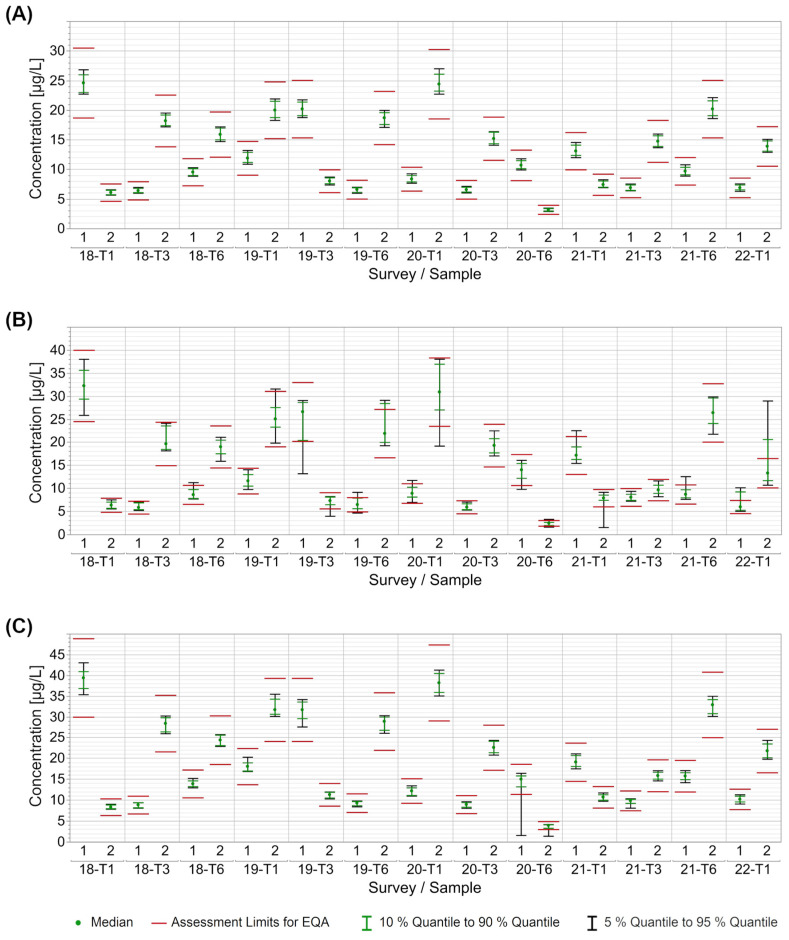
Manufacturer-dependent analysis of EQA results for CEA in relation to the current assessment limits set by RiliBÄK for the collectives RO (**A**), AX (**B**), and AB (**C**). The green dot represents the median of all results for the respective collective and EQA survey. The red lines indicate the assessment limit of ±24%, while the green lines indicate the median 80% of results and the black line the median 90% of results.

To gain an impression of the performance quality of the INSTAND EQAs for CEA and AFP detection between 2018 and 2021, we examined the middle 90% (5th to 95th quantile) and middle 80% (10th to 90th quantile) of the collective results in relation to the EQA assessment limit of a ±24% deviation from the target value in accordance with the RiliBÄK [18]. When looking at CEA surveys between 2018 and 2021, interval charts are shown exemplarily for the RO, AX, and AB collectives (Figure 3). The middle 90% of values of the RO collective lay well within the assessment limits for each sample (Figure 3A). In contrast, the middle 90% of the AX collective exceeded the range in about half of the samples, being sometimes above and sometimes below, which reflected a higher intra-assay variability. For survey 2022-T1, even the middle 80% of the EQA results exceeded the +24% assessment limit (Figure 3B). The AB collective performed comparably well to the RO collective. Only for samples in EQA 2020-T6 did individual participants in the AB collective report distinctly lower CEA values than other participants, resulting in a deviation of the middle 90% below the lower assessment limit (Figure 3C). The overall performance of the other reagent collectives was also similar to that of the RO collective, with the exception of individual surveys or samples (Figure S3A–C). The EQA performance of AFP detection was almost equivalent to CEA. The mean 90% of manufacturer-based results extended beyond the assessment limits only in the case of individual surveys or samples (Figure S4A–E). Only in one particular EQA (2020-T6) were a number of AFP results outside the lower assessment limits for several manufacturers (Figure S4).

## 4. Discussion

Cancer is a major global health problem. In 2020, there were more than 18 million new cancer cases worldwide (resulting in an age-standardized rate (ASR) of 190/100,000 person years). More than 10% of these new cases were CRC (ASR 19.5/100,000 person years), while 5% were liver cancer (ASR 9.5/100,000 person years) [21]. 

Tumor biomarkers play an important role in detecting and managing cancer. While only a few markers are suitable for screening, many established markers are beneficial for follow-ups (reviewed in [22]). CEA is only recommended as a monitoring marker for tumor entities such as CRC [23]. A metanalysis by Nicholson et al. concluded that the marker has a low sensitivity and is therefore not suitable for use as a single marker in the diagnosis of CRC [6]. However, it is frequently used as an additional diagnostic tool in the sensitive detection of recurrent or progressive disease in cancer surveillance after primary therapy. AFP, on the other hand, is not only recommended by several guidelines for monitoring liver cancer [24,25], but it also increases the chance of detecting HCC early on in patients with cirrhosis or chronic hepatitis C [2] when assessed serially alongside liver ultrasounds [7]. However, there have been studies for new tumor markers for HCC that showed a higher sensitivity and specificity for diagnosis. One recent meta-analysis by Guan et al. [26] shows a high sensitivity and specificity, especially for early-stage HCC, for the GALAD score, which includes gender, age AFP-L3, AFP, and des-gamma carboxyprothrombin (DCP) [27]. This score is already recognized by the FDA [28]. Other promising markers could show promising results as well, but the current studies included only small cohorts, so further research is needed [29,30]. In addition to its role as a tumor marker, AFP is also used as a maternal serum marker to detect neuronal birth defects [31] as part of a panel with other markers. The method recently demonstrated good test results in an EQA scheme [32].

The scientific community has been aware of manufacturer-dependent differences in the detection of the marker concentrations of CEA and AFP in serum for over 20 years [10,11,12,15,16,19,33]. The largest differences of 23% for AFP and 85% for CEA were found by INSTAND [13].

In this study, we re-evaluated new EQA data obtained between 2018 and 2022 for both markers to assess the scattering of results and current level of harmonization. We also analyzed the value distribution in accordance with the current German assessment limits set by the German Medical Association [18]. 

For CEA, we generally observed a maximum bias of 50% between the different manufacturer collectives with the exception of the TH collective, which showed notably higher results than all the other manufacturer collectives. The overall manufacturer-dependent differences were lower for EQA samples, with median CEA values below 20 µg/L. In the case of AFP, the manufacturer-dependent bias was lower than 20% for many EQA surveys, but we observed a bias of over 40% for individual EQA samples. Noticeable is the observation that a multimodality occurred in the SI collective in some EQAs for both markers. SI has taken over several other systems over the last few years and while the BG and the SIE sub-collective align quite nicely, the DG sub-collective has a tendency for higher values in the case of CEA detection (Figure S1 and Figure 1B) and lower results for AFP detection (Figure S2 and Figure 2B). In the case of AFP, the differences in the DG results have become more notable since October 2020 and they are especially remarkable in samples with higher AFP concentrations. Since an IRP is available for both markers [9], the manufacturers should be able, and in fact must be able, to align the calibration of their systems despite the different detection methods and systems.

For both markers, the median results reported by the RO collective did not differ much from the overall median of all results, as it had the largest number of participants contributing to the overall collective. Opposing trends in the relative medians of the individual collectives observed for individual EQA samples might be for several reasons, such as interfering substances or matrix effects.

In contrast to the observed inter-collective differences, the evaluated test systems showed a high within-method agreement of the reported values, with CVs mostly below 10% or even 5% in a few cases. 

Individual exceptions with high CVs can be observed. Such exceptions might be due to two possible reasons.

The first one would be due to different assay lots that might even be differently calibrated. According to Kim et al., this can have an effect on immunological tests. They found a relative reagent-based lot-to-lot variance of 0.1% to 17.5% for AFP [34]. The second reason could be that at least one of these assays could have had an issue with an interfering substance in the specific EQA samples. Immunoassays are prone to interfering substances, like immunoglobins, proteins, or lipids—so-called endogenous interfering substances—which can be derived from the donor (reviewed in [35]). The interference of non-specific cross-reactions, heterophilic antibodies, and paraproteins leading to falsely high or low tumor marker results must also be considered [36]. Three exceptions of high CVs were observed for the DG sub-collective in AFP detection that can be explained by high standard deviations that seems to be due to a combination of low numbers of participants and low mean values.

The observed robustness of most collectives is further underlined by the high passing rates of the participating laboratories (Figure 3, Figures S3 and S4). In most EQA surveys, the results of all participants were within the assessment limits set by the German Medical Association’s RiliBÄK guideline [18] as long as the evaluation is carried out using collectives. Our data showed that all results were often easily within the appropriate assessment limits of ±24% around the target value. However, there are differences between the collectives and, for CEA, the participants using the TH platform struggled more to stay within the acceptance range.

Both CEA and AFP are used for monitoring purposes. In the case of AFP, a reduction of more than 50% of the basal concentration after four weeks following localized concurrent chemoradiotherapy is a positive predictor for the effectiveness of the therapy in patients with progressed HCC [37]. For CEA, the National Academy of Clinical Biochemistry in the UK states that an elevated postoperative CEA concentration in serum of more than 30% should be considered significant; however, this increase should be reconfirmed by a second measurement before taking further diagnostic steps. Furthermore, the 30% change in concentration is more of a guidance and has yet to be clinically validated [38]. Other studies have found that small increases of 15% to 20% over three or more successive measurements may also indicate the need for further intervention (reviewed in [39]). There have been discussions on using biomarker reference change values (RCVs) to make clinical decisions [40,41,42,43,44]. The RCV is calculated on the basis of the within-subject biological variation and the analytical variation [42]. EQA data can be a helpful tool for gaining an impression of the current analytical variation of the test system used by each laboratory. 

Given that the current assessment limits in Germany lie with in a broad range of 48%, the discussion of narrower assessment limits would be desirable to further enhance the current quality of patient care. More stringent assessment limits could potentially avoid misdiagnosis and unnecessary, sometimes invasive, tests that put patients at risk. This has already been discussed in the case of HbA1c measurements (reviewed in [45]). A reduction in marker concentrations that have been determined to be too high or even, in the case of CEA, false positive results, is also beneficial for patient health, since abnormal values can cause incorrect treatment decisions, unnecessary invasive procedures, diagnostic radiation exposure, or, at minimum, mental harm to the patient [46,47].

Taken together, we observed a high within-method agreement for both markers. The currently observed manufacturer-dependent bias is lower for both markers compared with our previous publication [13], with a few exceptions. However, despite the fact that established IRPs for both markers have been around since 1975 [9], we still found a better harmonization for AFP results than for CEA. This could be due to several factors. 

First of all, CEA has a higher molecular weight than AFP and a higher carbohydrate content [48,49]. Additionally, several isoforms have been described for CEA [50]. Moreover, a higher number of epitopes has been reported for CEA [51] than for AFP [49]. These structural differences between both molecules might affect the definition of appropriate specific peptide epitopes for antibody binding of the immunoassays. In addition, the affinity of antigen–antibody binding may vary depending on the antibodies used in the assay as well as the conformation and glycosylation of their epitopes [52,53,54,55,56]. 

Furthermore, antibody characteristics can impact the binding specificity, which is likely to be lower for polyclonal antibodies than for monoclonal ones [57]. For the detection of AFP, we identified only assays that used monoclonal antibodies. During the period studied, all measurement systems for quantifying CEA were based on a reaction with monoclonal antibodies, except for the Advia system of SI, which used polyclonal antibodies. Contrarily, the binding of monoclonal antibodies is more susceptible to interfering substances, which may impair test sensitivity. Today, the interference of certain assay components, such as biotin and dyes like HABA, is suppressed in most assays, e.g., through the addition of corresponding blocking agents [58].

Based on the information in the test manuals, not all tests for detecting CEA that were used in the observed period of time were traceable to WHO standard 73/601, while all analyzed tests for AFP were traceable to WHO standard 72/225. As traceability to standard material is crucial for the harmonization of test-dependent EQA results, these differences in traceability of the various tests may explain the lower scatter of results for AFP than for CEA detection.

Additionally, both the current IRPs were established several years ago, and commutability of the material has likely not been verified. WHO standard 72/225 for AFP is derived from cord serum and the sugar chain structure of this cord serum AFP has been shown to differ from AFP secreted from HCC [59]. Since the binding epitopes of the test-specific antibodies are unknown, it is not possible to determine whether this different sugar chain structure might also contribute to the manufacturer-dependent differences observed in this study. 

In the case of CEA, Zhang et al. found varying potency of 73/601 in different buffer systems, which could indicate a matrix effect. They were also able to demonstrate that different test systems measured varying potencies for specific CEA standards from manufacturers other than the one producing the test system [15]. Furthermore, no harmonization could be observed in the detection of thyroid-stimulating hormone (TSH), even though the analyzed assays confirmed their traceability to the corresponding WHO IRP [60]. Taken together, since both reference preparations were established several years ago and under different ‘state-of-the-art’ procedures, it could be beneficial to establish new, commutable ones to further enhance the harmonization of assays for AFP and CEA. 

Another factor that must be taken into account is the commutability of the EQA materials used. Our samples consisted of serum from human donors. Where necessary, the samples were spiked with material derived from three-dimensional cell culture. The spiking of analytes is a common practice and, while the commutability of spiked samples could be confirmed for some enzymes [61] and β_2_-microglobulin, spiked carbohydrate-deficient transferrin proved to be non-commutable in another analysis [62]. Investigations are needed to determine whether and how much influence the spiking has on the EQA results. Nevertheless, manufacturer-dependent differences could also be observed within patient samples by several other researchers [10,11,15,16], so our observed manufacturer-specific results are most likely not caused by the spiked material alone. Other EQA providers also used spiked sample materials [12,33] and especially Sturgeon et al. deemed that a lack of commutability was unlikely due to the minimal manipulation of the sample material [19]. INSTAND is planning on conducting commutability testing for several tumor markers to address this issue for currently available tests. 

Our study shows that, until further harmonization is achieved, measurement systems should not be changed during tumor marker monitoring. In order to avoid misdiagnosis and unnecessary diagnostic treatment, labs should inform physicians about both the test results and the methods and measurement system used [7,8]. 

## 5. Conclusions

We found that the current assays for detecting AFP and CEA showed an overall better harmonization than previously reported. The assays of different manufacturers showed a good robustness and low intra-assay variation, making a further narrowing of current assessment limits in EQA schemes possible. This could stimulate further quality improvements in laboratory testing and result in a safer use of the changes in tumor marker values in the clinical guidance for cancer patients.

## Data Availability

All data generated or analyzed during this study are included in this published article and its Supplementary information files.

## Supplementary Materials

The following supporting information can be downloaded at: https://www.mdpi.com/article/10.3390/diagnostics13122019/s1, Figure S1: Analysis of EQA results for CEA levels for the heterogenous SI collective, shown from 2018 to the beginning of 2022. Data of two samples per survey are shown. The different red colored boxes display the results for the three main SI manufacturer sub-collectives BG, DG and SIE for the respective samples. The fourth SI collective has very low numbers of results and was therefore exempt from this analysis. For all boxes, the whiskers stretch from the 1st quartile − 1.5 * (interquartile range) to the 3rd quartile + 1.5 * (interquartile range). Outliers are not shown. Figure S2: Analysis of EQA results for AFP levels for the heterogenous SI collective, shown from 2018 to the beginning of 2022. Data of two samples per survey are shown. The different red colored boxes display the results for the three main SI manufacturer sub-collectives BG, DG and SIE for the respective samples. The fourth SI collective has very low numbers of results and was therefore exempt from this analysis. For all boxes, the whiskers stretch from the 1st quartile − 1.5 * (interquartile range) to the 3rd quartile + 1.5 * (interquartile range). Outliers are not shown. Figure S3: Manufacturer-dependent analysis of EQA results for CEA in relation to the current assessment limits set by RiliBÄK for the collectives SI (A), BE (B) and TH (C); Figure S4: Manufacturer-dependent analysis of EQA results for AFP in relation to the current assessment limits set by RiliBÄK for the collectives AB (A), SI (B), AX (C), BE (D) and RO (E); Table S1: CEA and AFP EQA results between 2018 and 2022 (T1) Raw data, Outliers that are most likely due to sample swaps, false magnitude (false unit) are marked in red font. Outliers as defined by jmp 16.0.0 from SAS Institute (Cary, North Carolina, USA) are marked in green font. In case of Siemens, jmp outliers were labled as jmp (SI) for the total SI collective (see Figure 1A and Figure 2A) or as jmp (BG), jmp (DG) and jmp (SIE) for the SI sub-collectives (see Figures S1 and S2); Table S2: Overview to the collectives considered in the EQA data analysis and corresponding information reported by the participants on the reagents and devices used.
