# Supplementary figures and images for: Longitudinal Evaluation of AFP and CEA External Proficiency Testing Reveals Need for Method Harmonization
Source: Diagnostics (Basel). 2023 Jun 9;13(12):2019. doi: 10.3390/diagnostics13122019 (PMC10296933; doi:10.3390/diagnostics13122019)

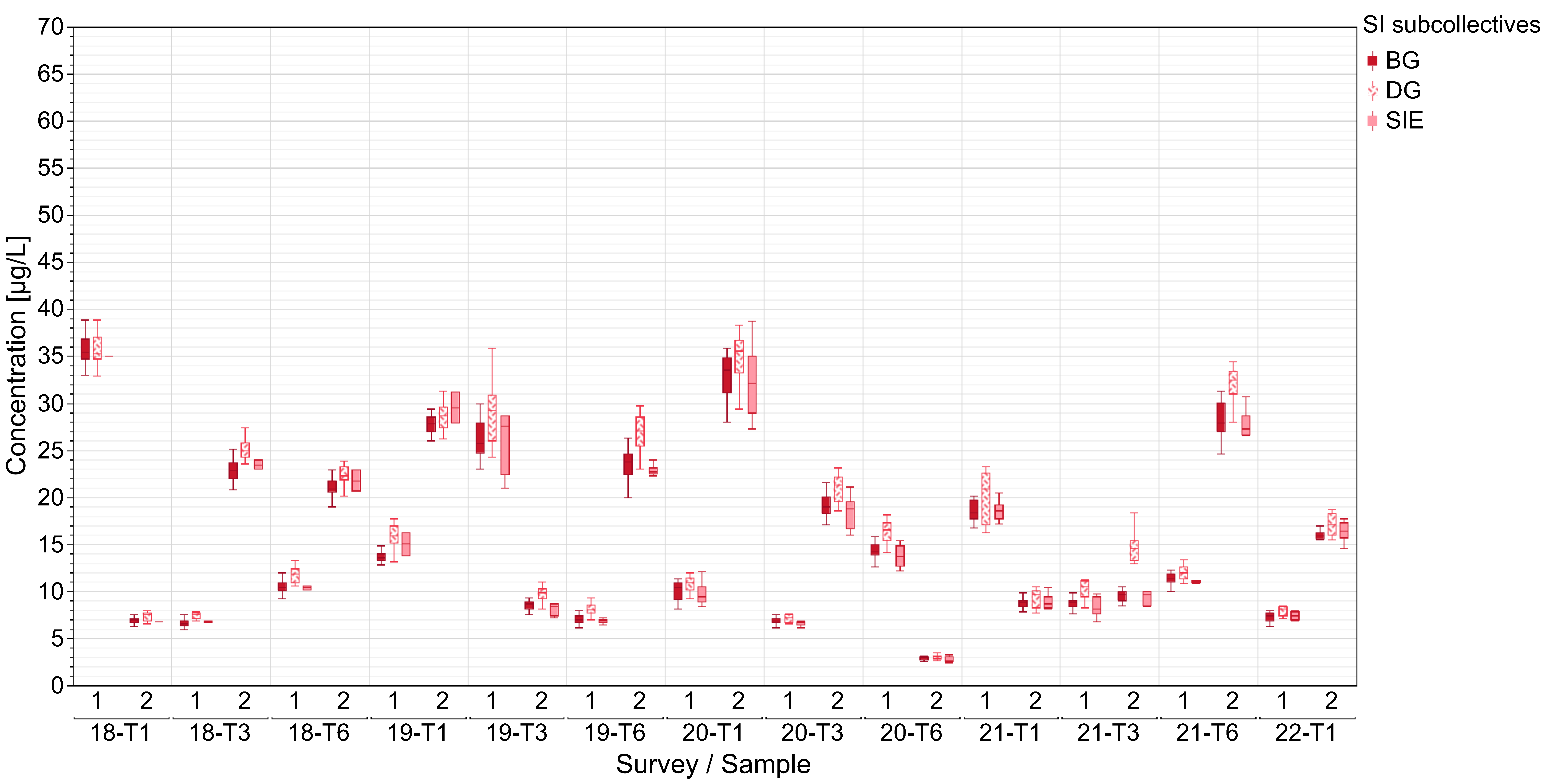

Supplement: Supplementary file 1 [file diagnostics-13-02019-s001.zip › Figure S1 CEA_SI subcollectives.tiff]

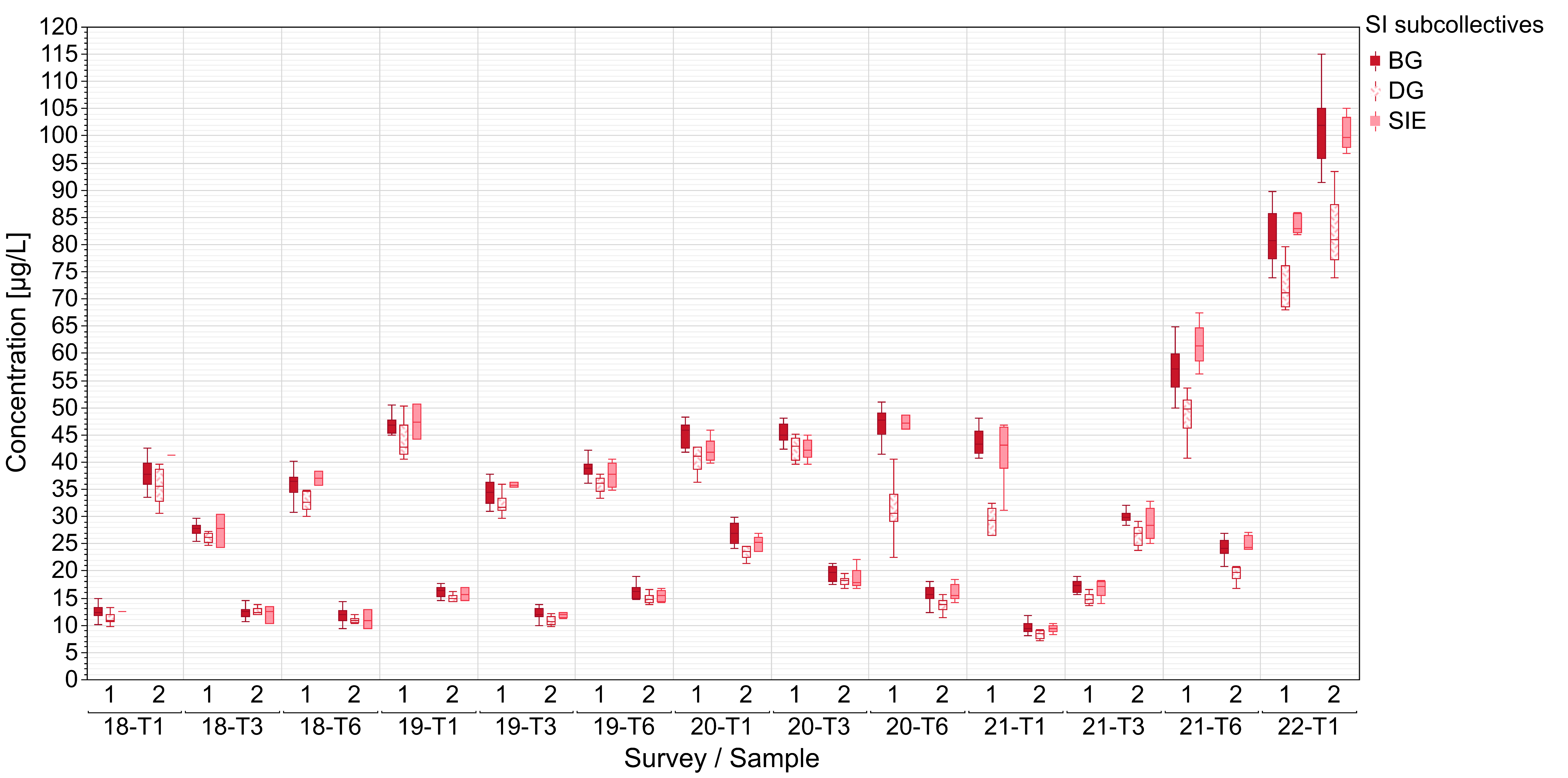

Supplement: Supplementary file 1 [file diagnostics-13-02019-s001.zip › Figure S2 AFP_SI subcollectives.tiff]

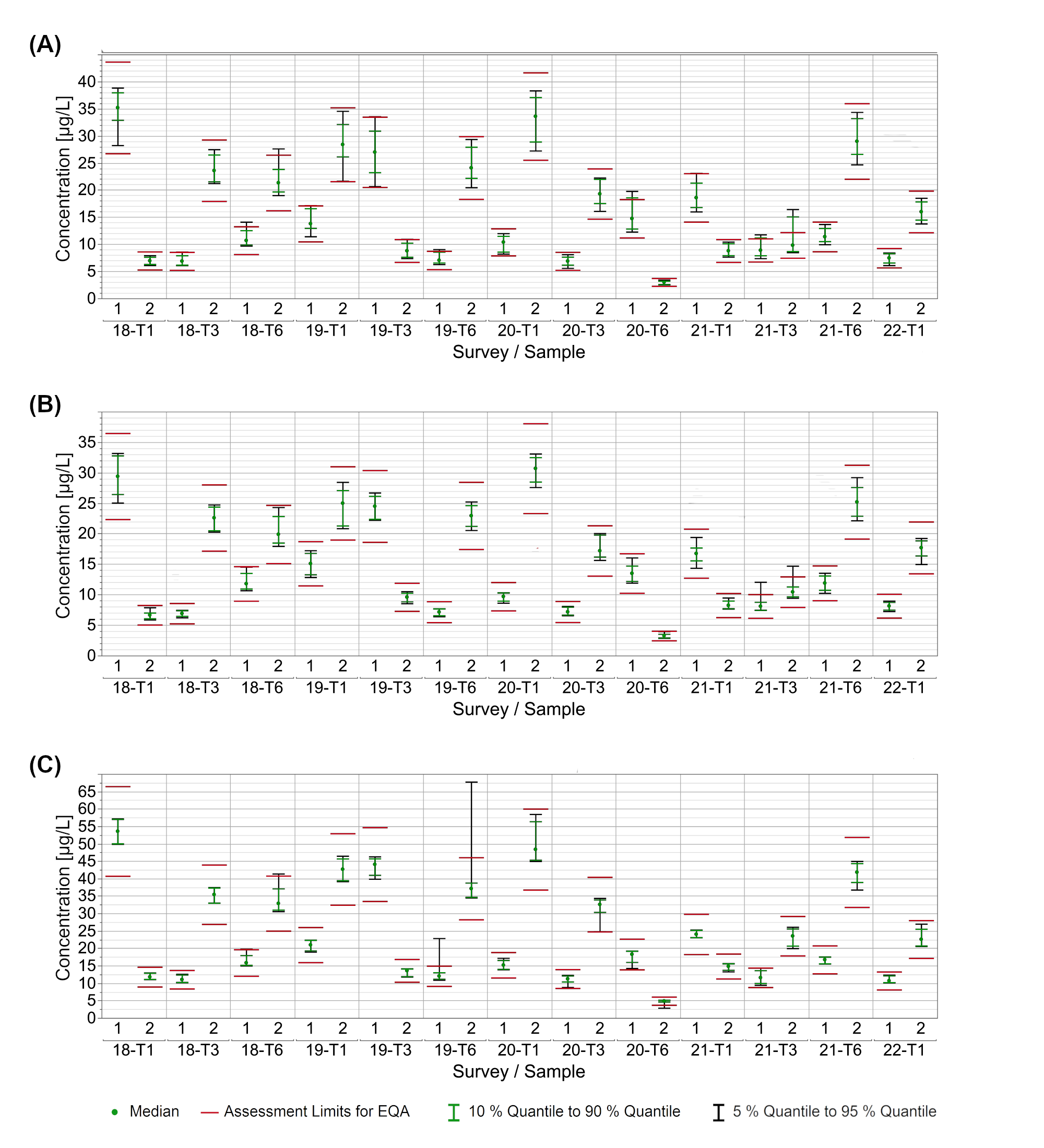

Supplement: Supplementary file 1 [file diagnostics-13-02019-s001.zip › Figure S3 CEA.tiff]

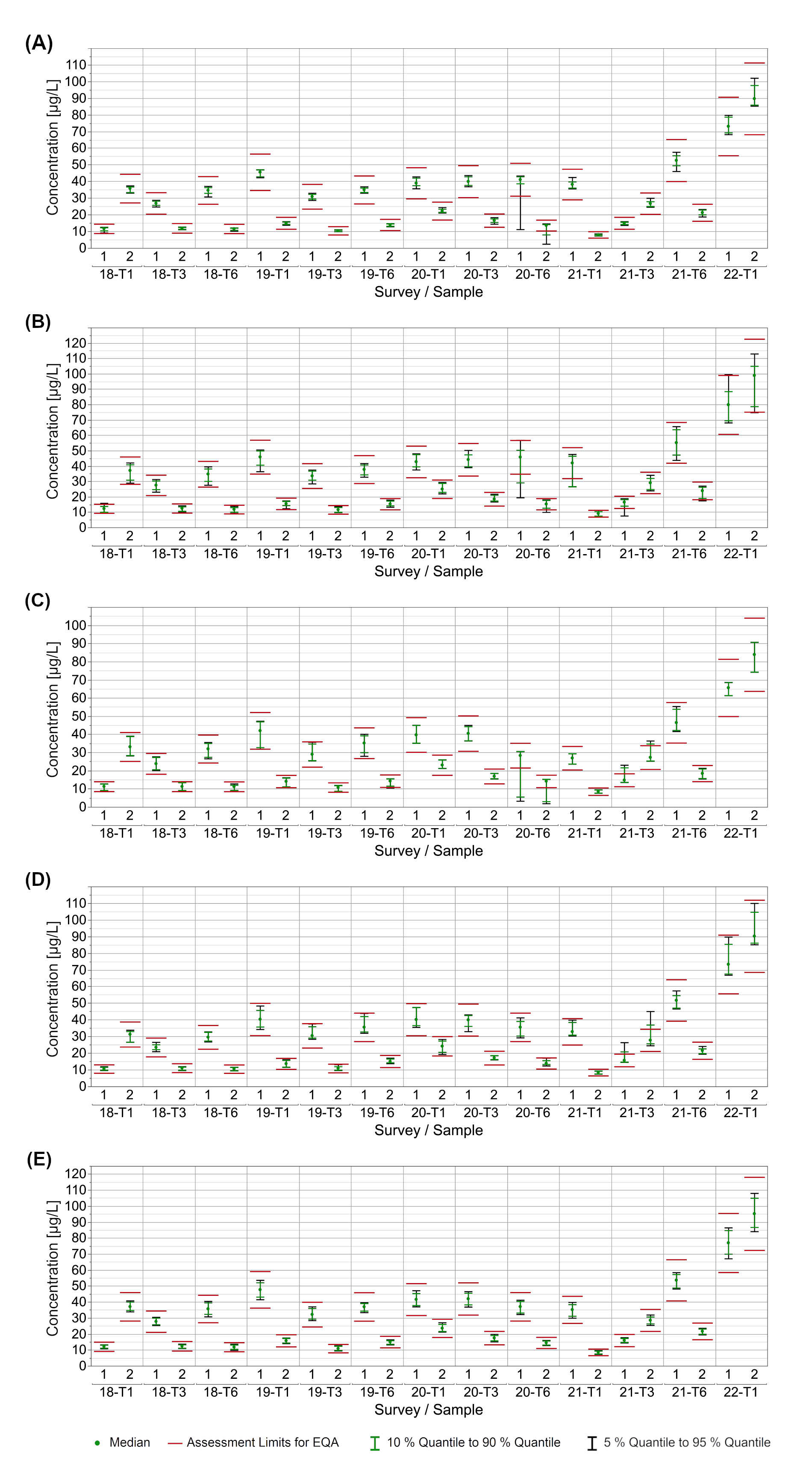

Supplement: Supplementary file 1 [file diagnostics-13-02019-s001.zip › Figure S4 AFP.tiff]
